# Ubiquitin-specific peptidase 37: an important cog in the oncogenic machinery of cancerous cells

**DOI:** 10.1186/s13046-021-02163-7

**Published:** 2021-11-10

**Authors:** Ravi Chauhan, Ajaz A. Bhat, Tariq Masoodi, Puneet Bagga, Ravinder Reddy, Ashna Gupta, Zahoor Ahmad Sheikh, Muzafar A. Macha, Mohammad Haris, Mayank Singh

**Affiliations:** 1 Department of Medical Oncology (Lab), All India Institute of Medical Sciences, New Delhi, India; 2 Laboratory of Molecular and Metabolic Imaging, Cancer Research Department, Sidra Medicine, Doha, Qatar; 3 Department of Genomic Medicine, Genetikode, Mumbai, India; 4 Department of Diagnostic Imaging, St. Jude Children’s Research Hospital, Memphis, TN USA; 5 Center for Advanced Metabolic Imaging in Precision Medicine, Department of Radiology, Perelman School of Medicine at the University of Pennsylvania, Philadelphia, USA; 6 Department of Surgical Oncology, Sher-I-Kashmir Institute of Medical Sciences, Srinagar, Jammu and Kashmir India; 7 Watson-Crick Centre for Molecular Medicine, Islamic University of Science and Technology, Pulwama, India; 8 Laboratory Animal Research Center, Qatar University, Doha, Qatar

**Keywords:** Ubiquitin, Deubiquitylating enzymes, Ubiquitin-specific peptidase, Ubiquitin-specific peptidase 37, Oncogene, Epithelial–mesenchymal transition

## Abstract

Protein ubiquitination is one of the most crucial posttranslational modifications responsible for regulating the stability and activity of proteins involved in homeostatic cellular function. Inconsistencies in the ubiquitination process may lead to tumorigenesis. Ubiquitin-specific peptidases are attractive therapeutic targets in different cancers and are being evaluated for clinical development. Ubiquitin-specific peptidase 37 (USP37) is one of the least studied members of the USP family. USP37 controls numerous aspects of oncogenesis, including stabilizing many different oncoproteins. Recent work highlights the role of USP37 in stimulating the epithelial-mesenchymal transition and metastasis in lung and breast cancer by stabilizing SNAI1 and stimulating the sonic hedgehog pathway, respectively. Several aspects of USP37 biology in cancer cells are yet unclear and are an active area of research. This review emphasizes the importance of USP37 in cancer and how identifying its molecular targets and signalling networks in various cancer types can help advance cancer therapeutics.

## Background

Cancer is characterized by the complex evolution of a healthy cell to a cancerous cell in which the gradual accumulation of mutations provides a survival advantage for growth and nutrition. Douglas Hanahan and Robert Weinberg first described the hallmarks of cancer in 2000 and later updated them in 2011 [1, 2]. These hallmarks of cancer comprise evading apoptosis, sustaining angiogenesis, being insensitive to antigrowth signals, developing limitless replicative potential, reprogramming energy metabolism, evading immune responses, acquiring genome instability, and promoting inflammation. They characterized the complexity of cancer and emphasized that treatment failure is related to unknown facets of cancer biology that drive the uncontrolled growth of cancerous cells. Because of advances in research methodologies and the emergence of new technologies, multiple factors controlling cancer cell evolution are being discovered, and posttranslational modifications of oncoproteins have emerged as an important factor for cancer cell evolution. These protein modifications include ubiquitinylation, Phosphorylation etc. which often occur in response to extracellular stimulus and reversal of these modifications also happens rapidly on the removal of stimulus. Ubiquitination refers to the covalent attachment of a 76 aa peptide to substrate proteins that control the half-life of proteins in a cell, coordinating the cellular localization of proteins, activating and inactivating proteins modulating protein-protein interactions. This is arguably one of the most important posttranslational modifications in cancer biology [3, 4]. The ubiquitination/deubiquitination cycle of proteins is synchronized to maintain cellular homeostasis.

Ubiquitin is a 76-amino acid (8.6 kD) peptide derived from four genes encoding mono or polyubiquitin chains. *UBA52* and *RPS27A* encode a single copy of ubiquitin fused to the ribosomal proteins L40 and S27a, whereas *UBB* and *UBC* encode polyubiquitin precursor proteins [3, 4]. Ubiquitination occurs at different sites on proteins, particularly at the lysine residues K6, K11, K27, K29, K33, K48, K43 and K63. Ubiquitination is controlled by three classes of enzymes, which are termed ubiquitin-activating enzymes (E1), ubiquitin-conjugating enzymes (E2), and ubiquitin ligases (E3) [5]. Initially, ubiquitin is activated in an ATP-dependent manner resulting in the formation of a thioester bond between the C-terminus of ubiquitin and active site of ubiquitin-activating enzyme (E1), then further transfer of ubiquitin molecule from E1 to the active site of the ubiquitin-conjugating enzyme or E2 and finally the ubiquitin is transferred to a lysine residue of the target protein with the help of ubiquitin ligases (E3) [5]. Ubiquitinated substrates are tagged for degradation by the subsequent accumulation of additional ubiquitin moieties. The addition of at least four ubiquitin moieties linked through the K48 ubiquitin chain to the targeted protein is sufficient to promote recognition and degradation by the 26S proteasome [6]. Additional linkages, such as K11-linked chains, also play a role in protein degradation [7].

Distinct ubiquitin linkage and branching of these chains give rise to the “ubiquitin code,” which determines the fate of many cellular proteins and ultimately affects cellular physiology [8]. The prime function of ubiquitin modification is targeting damaged or improperly folded proteins for degradation by the ubiquitin-proteasome system (UPS) [9]. In a seminal study, Hershko et al. observed that an amidase is responsible for removing the ATP-dependent proteolytic factor 1, later identified as a ubiquitin, from its substrate [10]. Later, Pickart and Rose reported that deubiquitinating enzymes (DUBs) hydrolyze amide derivatives of the ubiquitin carboxy-terminus by recognizing ubiquitin moieties [11]. DUBs essentially reverse the process of ubiquitination to replenish the ubiquitin pool in cells. Currently, the DUB family consists of nearly 100 enzymes that remove ubiquitin from different specific substrates [12].

DUBs belong to a subset of the protein family called cysteine proteases and are further classified into five families: ubiquitin-specific proteases (USPs), ovarian tumor proteases (OTUs), ubiquitin C-terminal hydrolases (UCHs), Machado-Joseph disease protein domain proteases (MJDs), and Jab1/Mov34/Mpr1 Pad1 N-terminal+ MPN+ (JAMM) motif proteases [13]. USPs, OTUs, UCHs, and MJDs are cysteine-dependent proteases, whereas JAMM motif proteases are metal-dependent cysteine proteases [14]. Two new families of DUBs were recently discovered: the MINDY family, which is specific for K48-linked ubiquitin chains, and the ZUP1 family, which is typical for K63-linked ubiquitin chains, linked to pathways involved in genome maintenance [15, 16].

USPs represent the largest subfamily of DUBs, which stabilize multiple oncoproteins via deubiquitination in different cancers. Therefore, USPs are excellent pharmacologic targets for cancer treatment by disrupting oncoprotein stability and function. The potential of targeting DUBs in cancer was extensively reviewed by Harrigan et al. and Huang et al. [12, 17]. In the last decade, elucidating the role of many USPs, such as USP2, USP7, USP10, USP22, USP44, USP9X, and USP14, in different cancers led to the development of inhibitors for USP7, USP14, USP1, and USP9X [18]. However, understanding the role that many other USPs play in cancer is still in the infancy stage. New substrates and new mechanisms of action are now coming to light.

Here, we focus on USP37, first described by Huang et al. in 2011 [19]. We comprehensively discuss its involvement in many critical cellular processes, such as the cell cycle, homologous recombination (HR), histone modifications, and oncoprotein stability, in multiple cancer types, including lung, breast, and kidney cancer. We also discuss the influence of USP37 on a myriad of signaling pathways and the potential of these pathways for novel therapeutic interventions in cancers.

## USP37 regulates cell cycle progression

Many DUBs are postulated to affect the cell cycle, and modulation of the cell cycle is a frequent mode of action of many oncogenes. USP37 levels fluctuate during cell cycle progression, accumulate in late G_1_/S, decrease in late mitosis, and reappear during G1. Huang et al. reported that in U2OS cells, USP37 influences cell cycle progression by regulating the expression of cyclin A [19]. Mechanistically It was seen that the E2F transcription factor activated USP37 transcription in late G1, Which results in accumulation of Cyclin A leading to G1/S transition. The buildup of cyclin A, a crucial regulator of the G1/S cell cycle transition, is delayed in U2OS cells that express USP37 shRNA [19].

**Fig. 1:**
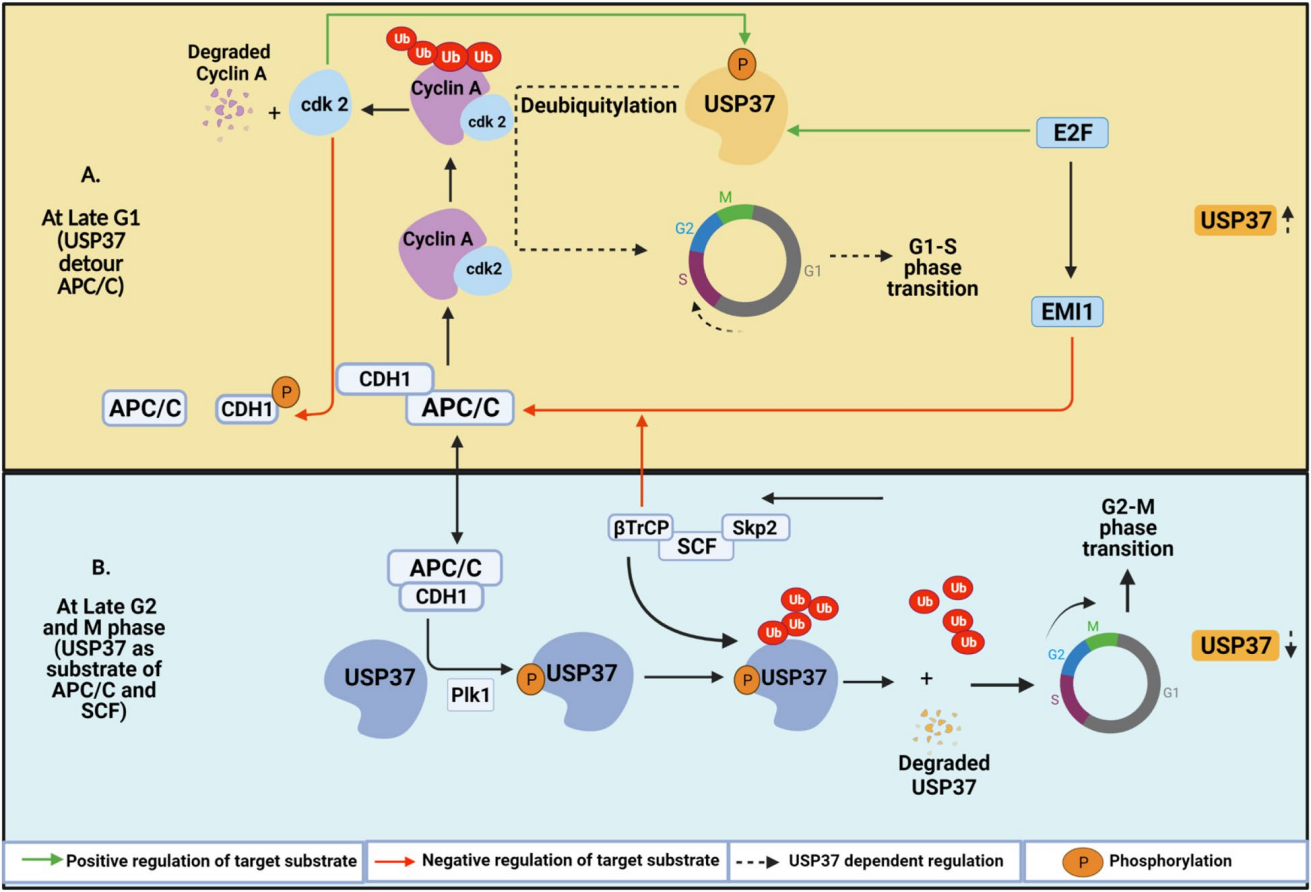
Model of USP37 switching in different stages of the cell cycle. USP37 levels fluctuate during cell cycle progression. The anaphase-promoting complex (APC) targets cell cycle proteins for proteasomal degradation through coactivators. **A** In the late G_1_ stage, USP37 expression is found to be elevated as it is being phosphorylated by cdk2 and stabilized by the E2F transcription factor. However, Cdk2 also phosphorylates a coactivator of APC Complex i.e., CDH1, which together with EMI1 negatively regulates APC complex resulting in the accumulation of USP37 that detours APC/C and stabilizes cyclin A through deubiquitination, required for G1/S phase transition. **B** In the late G2/M phase, USP37 acts as a substrate of the APC/C complex via phosphorylation by PLK1 and is further ubiquitinated by βTrCP for the biphasic degradation, resulting in the downregulation of USP37, necessary for the G2/M phase transition

The anaphase-promoting complex (APC) is an E3 ubiquitin ligase that engages CDC20 and CDH1 as coactivators to target proteins for proteasomal degradation. Huang et al. characterized the USP37 interaction with CDH1 and CDC20 by tandem mass spectrometry and found that USP37 interacts with CDH1 but not CDC20. The core components of APC bind with USP37 through CDH1. Accumulation of APC complex substrate E2F transcription factor targets EMI1, which completely inhibits APC and induces USP37 in the G1 phase (Fig. 1). Cyclin A is a known substrate of the APC-CDH1 complex and it accumulates despite an active APC–CDH1 complex, suggesting a potential escape mechanism from proteasomal degradation. USP37 was postulated to directly bind and stabilize cyclin A by stimulating its deubiquitination, promoting its accumulation and progression to G_1_/S.

Interestingly, the APC–CDH1 complex degrades USP37 in late mitosis by targeting its KEN box degron. A mutation in the KEN box degron site affects the stability of USP37 and its ability to deubiquitinate cyclin A. The APC-CDH1 complex does not recognize USP37 as a substrate during S/G2, possibly due to phosphorylation of USP37 by cyclin-dependent kinase 2 (CDK2)/cyclin A. Deubiquitination and phosphorylation of CDH1 by CDKs dissociates CDH1 from the core APC complex during S/G2. In late mitosis, CDK2 is no longer active, and thus the APC–CDH1 complex reforms and dephosphorylated USP37 switches from an antagonist to a substrate of the APC-CDH1 complex (Fig. 1). CDH1 is a postulated tumor suppressor, and USP37 may thus act as a tumor suppressor in concert with CDH1. Therefore, the function of USP37 in different cancer types may be context-specific.

Sowa et al. reported the interaction of USP37 with SCF^βTrCP^/CUL1, βTrCP, and SKP1 [20]. In concordance, Burrows et al. reported cell cycle-dependent fluctuations of USP37 by the SKP1-CUL1-F-box (SCF) E3 ubiquitin ligase [21]. Biphasic destruction of USP37 occurs in G2 by the concerted action of Polo like Kinase (PLK1) and SCF^βTrCP^, APC–CDH1 targets the remaining pool during mitotic exit. The underlying mechanism involves PLK1 kinase-mediated phosphorylation of USP37 and its binding to βTrCP, promoting ubiquitination and degradation (Fig. 1). The SCF^βTrCP^ E3 ubiquitin ligase modulates many other cell cycle regulators, such as EMI1, WEE1, BORA, and claspin, promoting G2/M progression [22, 23]. In contrast, positive regulation of USP37 is associated with increased cyclin A/CDK2 activity and development of hepatocellular carcinoma (HCC) by the hepatitis B virus HBx oncoprotein [24]. The cyclin A–CDK2 complex hyperphosphorylates and inactivates pRB, activates the E2F transcription factor, and accelerates G_1_/S phase transition. However, HBx escorts USP37 from the nucleus, downregulates CDH1, and prevents its SCF^βTRCP^mediated degradation via increased CDK2-mediated phosphorylation [24]. The HBx oncoprotein induces cell proliferation by stabilizing cell cycle regulators such as cyclin A, reducing their ubiquitination. Together, these findings highlight the importance of USP37 for regulating cell cycle progression and how its dysregulation may drive tumorigenesis.

## USP37 as a stabilizer of oncoproteins

**Fig. 2:**
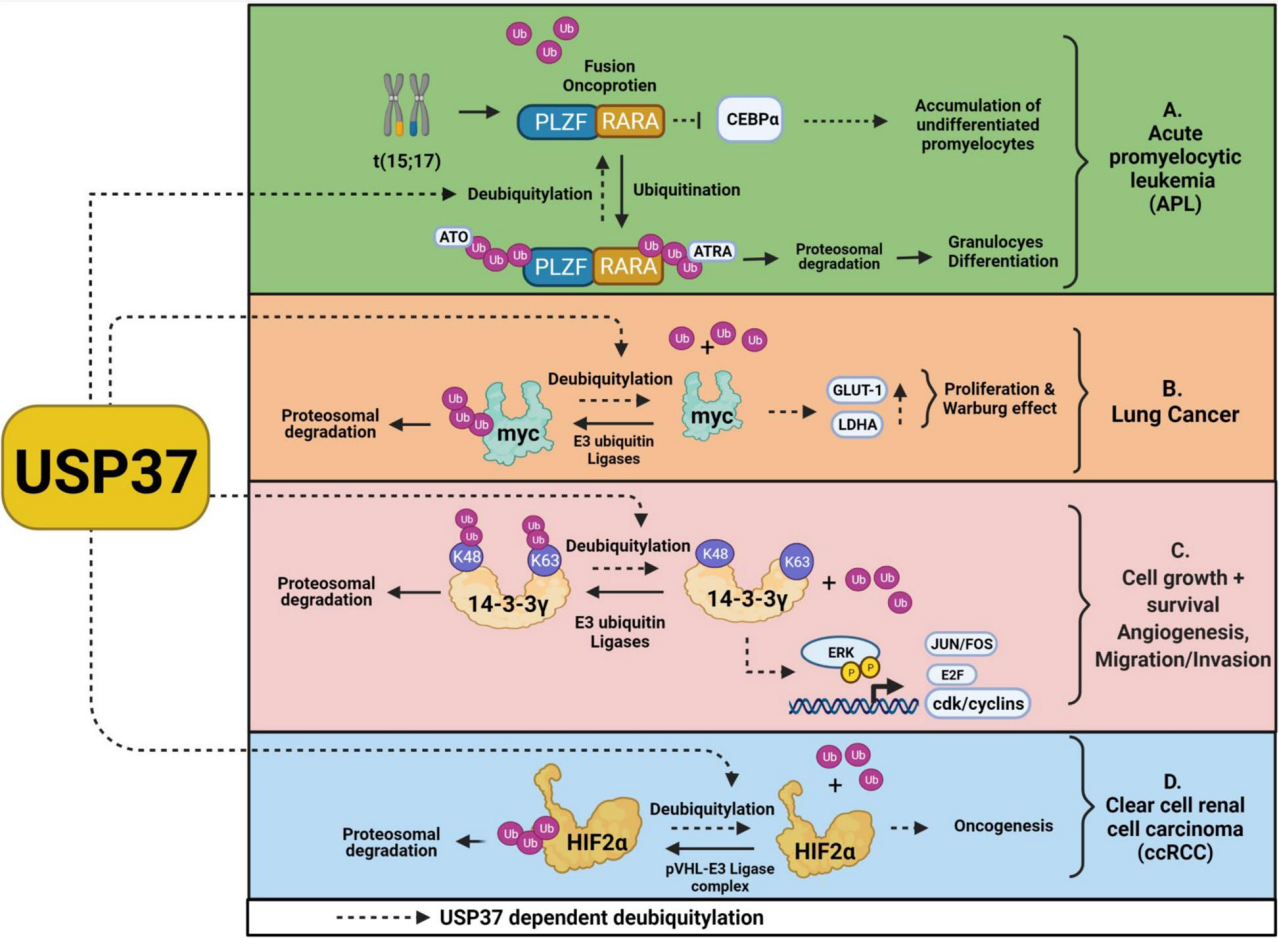
Overview of the role of USP37 in stabilizing various oncoproteins in different cancers. **A** USP37 interaction with the PLZF moiety of the PLZF/RARA fusion protein and its stabilization by suppressing the effects of arsenic trioxide (ATO) and all-trans retinoic acid (ATRA) treatment through deubiquitination and promoting cell transformation in APL. **B** USP37 direct interaction with c-MYC and its stabilization in a DUB activity-dependent manner for further regulation of cell proliferation and the Warburg effect in lung cancer. **C** USP37 interaction with 14–3-3γ and its stabilization via deubiquitination for further cell growth and proliferation. **D** USP37 interaction and stabilization of HIF2α and its involvement in oncogenesis in clear cell renal cell carcinoma

Many studies have conclusively established the role of USP37 in stabilizing various oncogenes and promoting tumorigenesis [25–28]. Acute promyelocytic leukemia (APL) is characterized by chromosomal translocations between the retinoic acid receptor alpha (*RARA or PARα*) gene and the promyelocytic leukaemia (PML) or promyelocytic leukaemia zinc finger (PLZF) genes among which PML-RARA is the most common gene fusion APL cells expressing the PLZF/RARα fusion protein are largely resistant to standard treatments and are associated with poor prognosis. RNAi-based screening identified many DUBs, including OTUD6A, OTUD7B, USP2, USP9, and USP37, that interact with the PLZF/RARα fusion protein and increase its stability [25]. Interestingly, PLZF but not RARα levels are elevated in response to USP37 overexpression. Furthermore, USP37 interacts with a PLZF moiety and stabilizes the PLZF/RARα fusion protein (Fig. 2A). These discoveries were the first to identify the USP37-mediated modulation of PLZF/RARα stability and development of APL.

The c-MYC oncogene is regulated by many ubiquitin ligases, such as F-box and WD repeat domain-containing 7 (FBW7), SCF^βTrCP^, and SKP2 [29–31]. In addition, the DUB USP28 stabilizes c-MYC expression. To investigate the molecular landscape of DUBs that affect c-MYC activity, Pan et al. expressed various USPs in HEK293T cells and observed that USP37 alters endogenous c-MYC levels. Furthermore, USP37 deubiquitinates c-MYC in lung cancer H1299 cells and drives proliferation and the Warburg effect [26]. Immunohistochemical analysis revealed that USP37 is upregulated in 64% of human lung cancer tissues and is associated with c-MYC expression [26]. Recently, hypomethylation of the *USP37* promoter was found to be associated with its increased mRNA levels in lung adenocarcinoma and squamous cell carcinoma as well [32]. Therefore, USP37 is a potent oncogene that drives the expression of the c-MYC oncoprotein and lung cancer oncogenesis (Fig. 2B).

Overexpression of the 14–3-3γ oncoprotein is associated with the invasion and metastasis of cancer cells [33–35]. Moreover, 14–3-3γ inhibits apoptotic cell death and promotes cell proliferation in immune cells. Proteomic and functional analysis of 14–3-3γ–binding proteins revealed that USP37 is a potential binding partner [36]. Subsequently, Kim et al. observed that USP37 regulates the stability of ubiquitin-conjugated 14–3-3γ by removing K48- and K63-branched chains [27], thereby increasing cell proliferation and potentially contributing to malignant transformation via the MAPK signaling pathway by regulation of pERK levels (Fig. 2C).

USP37 regulates the stability of hypoxia-inducible factor-2α (HIF2α) in clear cell renal carcinoma and is associated with loss of the tumor suppressor von Hippel–Lindau (VHL). VHL contains intrinsic ubiquitin ligase activity and is mutated in 70% of kidney cancers, leading to the accumulation of HIF2α factors [37]. After translocation to the nucleus, HIF2α dimerizes with the HIF2β subunit and transactivates genes involved in angiogenesis, glycolysis, glucose transport, and erythropoiesis [38]. Hong et al. used a FLAG- and HA-tagged DUB complementary DNA library containing 60 DUB genes to identify DUBs that bind to endogenous HIF2α, including OTUD7B/Cezanne, USP29, USP39, and USP37. Among these DUBs, USP37 induced the most significant increase in HIF2α levels (Fig. 2D). Functionally, downregulated USP37 decreases cell proliferation and anchorage-independent growth [28]. This was further validated in an orthotopic mouse model in which *USP37* depletion reduces primary kidney tumorigenesis and decreases lung metastasis. Therefore, USP37 drives oncogenesis by altering the stability of various oncoproteins and activating downstream signaling pathways.

## Modulation of the cancer stemness and drug sensitivity by USP37

Recent studies have conclusively established that USP37 stabilizes many oncoproteins involved in regulating various hallmarks of cancer. More recently, in a bioinformatic analysis of breast cancer (BC), The Cancer Genome Atlas (TCGA) database revealed upregulation of USP37 that is associated with increased mortality [39]. In addition, increased USP37 mRNA levels were observed in CD24^−^CD44^+^ BC stem cells compared to CD24^+^ or CD24^−^CD44^−^ cells (from BC MCF7 cells). Furthermore, USP37 was also elevated at the protein level in cancer stem cell spheroids compared to adherent cells, and its knockdown (KD) decreased expression of stem cell markers like smoothened, Gli-1, ALDH1, and OCT4 and inhibited stable spheroid formation. USP37 KD in MCF7 cells also increased sensitivity to chemotherapeutic agents such as cisplatin by decreasing the BCL2/BAX ratio and attenuating tumor growth in vivo [39]. It was seen that in adriamycin-resistant MCF7 cells (MCF7/ADR), *USP37* levels are increased and its KD promotes adriamycin sensitivity by decreasing the BCL2/BAX ratio [40]. These findings provide early evidence that USP37 modulates drug resistance. Targeting USP37 alone or in combination with other drugs may be an excellent approach to achieve synthetic lethality in cancer cells.

## USP37 alters DNA replication dynamics and the DNA damage response

The DNA damage response (DDR) is a safeguard mechanism to prevent errors in genome duplication and preserve genomic stability. The major pathways involved in DNA repair include Homologous recombination (HR) and non-homologous end-joining (NHEJ). DNA double-strand breaks (DSBs) elicit a signalling cascade initiated by the central DDR protein ataxia telangiectasia mutated (ATM) kinase, which accumulates the phosphorylated histone H2A variant H2AX. This cascade involves the E3 ubiquitin ligases RNF8/RNF168 and the ubiquitin-dependent assembly of the BRCA1–Abraxas–RAP80–MERIT40 (BRCA1-A) complex [41]. Deubiquitination of these complexes is an essential component of this cascade because it is required to fine-tune the DDR.

Typas et al. published one of the earliest reports of the role of USP37 in the HR pathway [42]. Using image-based genetic screens, they identified two ubiquitin-specific peptidases USP37 and USP26, as critical DUBs that limit the repressive effect of RNF8/RNF168 signalling on the HR pathway. USP37 and USP26 are recruited to DSBs to remove RNF168-induced ubiquitin conjugates from the BRCA1-A complex, whereas loss of *USP37* impairs DSB repair. USP37 and USP36 prevent excessive spreading of RAP80-BRCA1 from DSBs. This suggests that both USPs act in a concerted manner to limit the ubiquitin-dependent sequestration of BRCA1 by the BRCA1-A complex and simultaneously promote the complex formation of BRCA1 with the PALB2-BRCA2-RAD51 complex, thereby initiating the HR pathway. These findings are an elegant example of how two different DUBs coordinate to achieve a physiologic outcome that defines a cellular process.

Initiation of DNA replication in eukaryotes involves several factors (protein–protein complexes) and a tightly regulated replication machinery that controls changing levels of these factors throughout the cell cycles by implementing ubiquitination/deubiquitination processes [43]. The origin recognition complex is a hetero-hexamer with DNA-dependent ATPase activity that directly recognizes and binds replication origins. CCD6 and CDT1 are recruited to the replication origins, which load the minichromosome maintenance protein complex MCM2–7 with intrinsic ATPase-dependent DNA helicase activity onto replication origins. Helicase binding to DNA initiates the licensing of replication origins and forms the pre-replicative complex [44]. CDT1 is an essential factor during DNA replication whose levels are controlled by the ubiquitination machinery, which fluctuate with the cell cycle and in response to DNA damage [45–47]. Using an overexpression screen of 78 human ubiquitin and ubiquitin-like hydrolases, Perez et al. reported that USP37 increases CDT1 levels when cells are exposed to DNA-damaging agents while *USP37* depletion with siRNA decreases CDT1 protein levels [48]. Because *CDT1* mRNA levels are unchanged, USP37-mediated stability of CDT1 is controlled at the protein level. Interestingly, immunoblotting of CDT1 revealed that the protein migrates as two species, of which the larger species is phosphorylated, stabilized after *USP37* overexpression, and has increased affinity for USP37. USP37 induces deubiquitination of CDT1, whereas a catalytically inactive (C350S) *USP37* mutant is unable to ubiquitinate CDT1, further suggesting that USP37 plays a role in CDT1 stabilization. In *USP37*-depleted cells, the replication fork progression rate is delayed, but the percentage of replication fork firing increases. This suggests that other proteins might be modulating replication fork firing, which regulates cell survival after DNA damage, and USP37 functions as an oncogene.

## USP37 as a modulator of the epithelial-mesenchymal transition and metastasis

A study by Qin et al. reported that *USP37* KD suppresses BC and BC stem cell migration and invasion by promoting the mesenchymal-epithelial transition (MET) by markedly reducing the SNAI1, N-cadherin, and vimentin expression and increasing the E-cadherin. In contrast, *USP37* upregulation promotes EMT, migration, and invasion [39]. Further dissection of the underlying mechanisms of USP37-regulated EMT and MET indicated that USP37 participates in the sonic hedgehog (SHH) pathway and the stability of GLI1, a zinc finger transcription factor activated by ligand binding to the patched SHH receptor [39, 49]. Therefore, USP37 promotes EMT via the SHH pathway, and the signalling outcomes are determined by the balance of activated and inhibitory GLI1 proteins [39].

Two parallel studies in 2019 and 2020 highlighted the role of USP37 in inducing EMT by modulating the SNAI1 protein. SNAI1 is an EMT-inducing zinc finger transcription factor that promotes EMT-mediated metastasis by binding to E-boxes, thereby repressing the expression of a large pool of genes that control epithelial identity and lead to the conversion of healthy epithelial cells to a mesenchymal cell phenotype [50, 51]. *SNAI1* expression is associated with chemotherapy resistance, reduced survival, poor prognosis, and relapse [52]. Interestingly, SNAI1 is a highly labile protein with a half-life of approximately 30 min [53] that is regulated by many E3 ubiquitin ligases, such as β-TrCP, FBXL14, FBXO11, and FBW7, which ubiquitinate and promote its degradation through the proteasomal complex [54–57]. SNAI1 is also deubiquitinated by at least three DUBs: DUB3, PSMD14, and OTUB1 [58–61]. Xiao et al. screened SNAI1-interacting DUBs with a panel of 68 tagged human DUBs. Each DUB was cotransfected with MYC-tagged SNAI1 in HEK293T cells [62]. Subsequent sub-screening identified 23 DUBs that interact with SNAI1, out of which USP29, USP36, and USP37 upregulate and reduce polyubiquitination of the SNAI1 protein. Of these three DUBs, USP37 binds SNAI1 most strongly and plays a major role in stabilizing SNAI1 and thereby promoting cancer cell migration, E cadherin downregulation, and vimentin upregulation. Indeed, *USP37* KD reduces cell migration in transwell assays [62]. Moreover, a catalytically dead *USP37* mutant cannot promote cancer cell migration, indicating that the DUB activity of USP37 is required for its metastatic properties.

A parallel study by Cai et al. reported similar observations in 293 T cells and lung cancer cell lines. They transfected plasmids encoding 79 DUBs into 293 T cells expressing Flag-SNAI1 and found that USP37 predominantly interacts and stabilizes SNAI1 by causing its deubiquitination. They further validated these findings in lung cancer H1299 cells [63]. Furthermore, expression of wild-type but not a catalytically dead mutant of *USP37* induces a migratory phenotype in lung cancer cells.

**Fig. 3:**
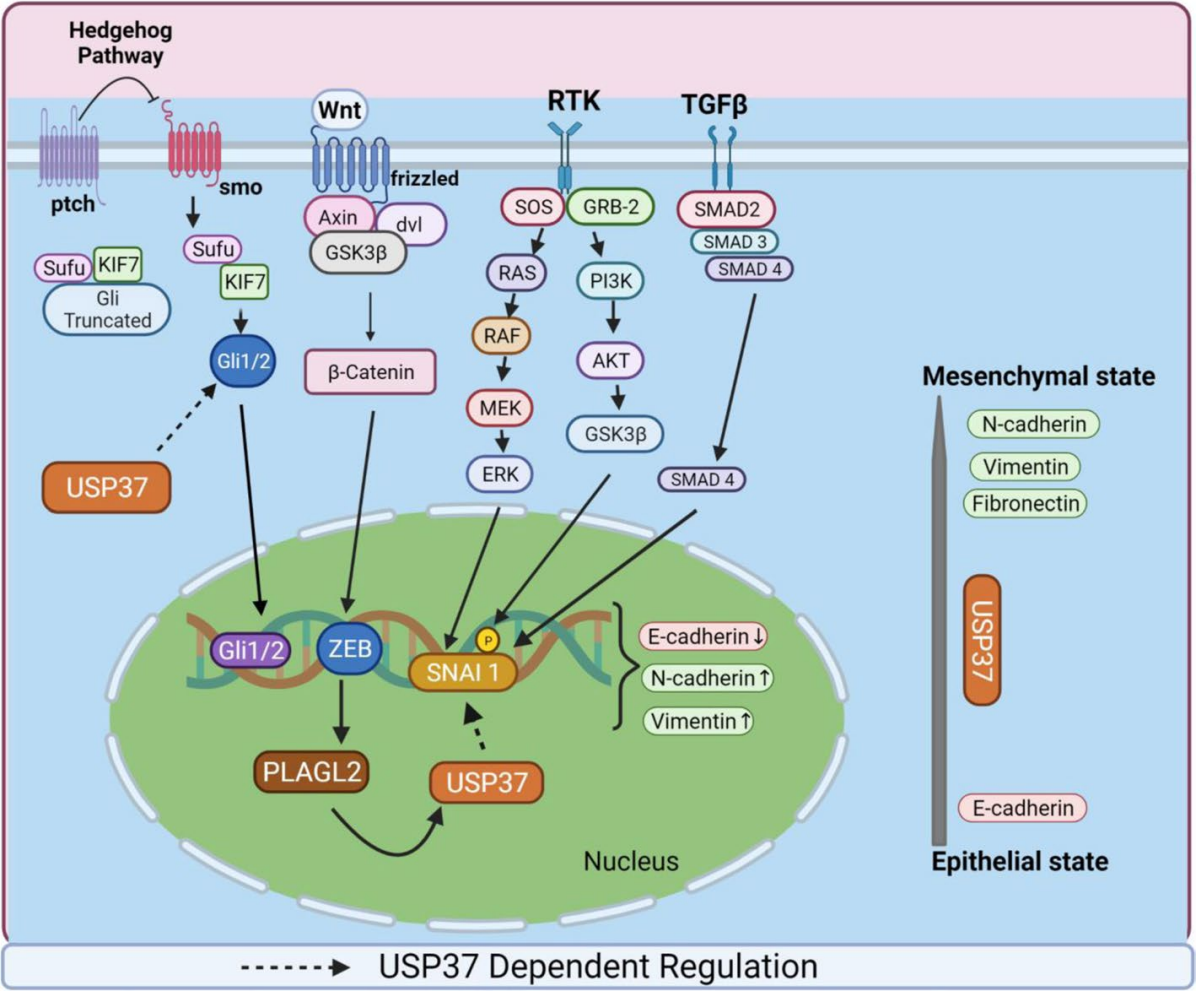
USP37 is a modulator of the epithelial-mesenchymal transition and metastasis. Illustration of the regulation of EMT and metastasis by USP37 via its interaction with components of the SHH pathway (GLI1) and deubiquitination of SNAI1 via PLAGL2

A recent study by Wu et al. reported the detailed mechanism of USP37-mediated deubiquitination of SNAI1 in gastric cancer (GC) cells [64]. Pleomorphic adenoma gene like-2 (PLAGL2), a zinc finger PLAG transcription factor, is aberrantly expressed in several cancers, including GC, and binds to GRGGC(N)6-8RGGK consensus sequences in the promoters of its target genes. PLAGL2 also induces EMT and colorectal cancer metastasis by β-catenin–dependent regulation of ZEB1 and its associated signalling pathways [65, 66]. PLAGL2 promotes proliferation, migration, and invasion of GC cells by downregulation of E-cadherin and promotes tumor growth in vivo [64]. Depletion of *PLAGL2* with siRNA reduces SNAI1 protein levels in GC SGC7901 cells, whereas *PLAGL2* overexpression elevates SNAI1 protein levels in AGS cells. Importantly, *SNAI1* mRNA levels do not change, suggesting that PLAGL2 modulates SNAI1 expression post-transcriptionally [64]. Microarray analysis of total RNA isolated from stable PLAGL2 knockdown (SGC7901-shRNA) and SGC7901 NC cells elucidated three differentially expressed DUBs, of which only USP37 directly interacted and significantly diminished the ubiquitination of SNAI1. The serine-rich domain (SRD) within SNAI1 is required to interact with USP37 and is critical for its downstream signalling. Removal of phosphate groups from SNAI1 with a dephosphorylase enzyme reduces the interaction between USP37 and SNAI1; therefore, phosphorylation is required for their interaction. The phosphorylation of SNAI1 is mediated by GSK3β in the SRD region. However, a mutant *SNAL1*^6SA^ containing six putative GSK3β phosphorylation sites within the SRD domain markedly decreases its interaction with USP37. Finally, PLAGL2 modulates SNAI1 stability by activating USP37 transcription. PLAGL2 specifically binds to the GRGGC(N)6-8RGGK consensus sequences in the *USP37* promoter region and activates its transcription, subsequently stabilizing SNAI1 and promoting EMT in GC cells [64]. Therefore, USP37 influences EMT and cell metastasis by stabilizing SNAI1 and by modulating the SHH pathway in lung, gastric, and BCs (Fig. 3).

## Micro-RNA–based transcriptional regulation of *USP37*

Micro-RNAs (miRNAs) are small non-coding RNAs, approximately 22 nucleotides in length and regulate many oncogenic pathways by posttranscriptional modification of targets genes [67]. In most cases, miRNAs interact with the 3′ untranslated region (3′ UTR) of target mRNAs to induce mRNA degradation and transcriptional repression [68, 69]. Many DUBs are targets of miRNAs. Two recent studies revealed the interaction of miRNAs with *USP37* mRNA and its modification to regulate its levels.

In the first study, Qin et al. reported that miR-30B-5p targets and modifies *USP37* mRNA in HCC [70]. In many cancers, miR-30B-5P acts as a tumour suppressor and exerts an inhibitory effect on cell proliferation and the cell cycle [71, 72]. *DNMT3A*, a DNA methyltransferase, is targeted by miR-30B-5p to downregulate its expression and repress HCC cell proliferation. Intriguingly, this study also found that miR-30B-5p arrests cell cycle progression during S phase, possibly by regulating *USP37* expression. According to Targetscan prediction of miR-30b-5p targets, miR-30b-5p may have 3 binding sites on 3′ UTR of USP37 mRNA (position 674–681, 3057–3063, and 3131–3138). Mutating the binding site sequences of the *USP37* 3′ UTR revealed that miR-30B-5P binds (position 3057–3063) of *USP37* 3′ UTR, which was confirmed by a luciferase reporter assay and suggested that miR-30b-5p promote its degradation and thereby repress cell proliferation [70].

A second study by Li et al. revealed that miR320b acts as a tumor suppressor in lung cancer (LC) that is complicated by obstructive sleep apnea-hypopnea (OSAH) by regulating CDT1 via USP37 [32]. Patients with lung cancer and OSAH have an increased propensity of intermittent hypoxia in the upper lobes of the lungs, exacerbating LC tumorigenesis and progression [73, 74]. Expression of miR-320b is reduced in patients with lung cancer and OSAH and LC cells subjected to Intermittent hypoxia (IH). Furthermore, overexpression of miR-320b reduces the proliferation of LC cells exposed to IH. Analysis of USP37 protein expression with bioinformatics tools (e.g., microT and miRWalk) indicated that miR-320b and USP37 expression is reciprocally correlated. Because USP37 is highly expressed in patients with LC, OSAH and LC cell lines, USP37 may be involved in OSAH-associated lung cancer progression. Through prediction algorithm, the targeting sites of miR-320b on *USP37* was confirmed by a luciferase reporter assay at the 3′ UTR of *USP37*. Furthermore, miR-320b overexpression reduces IH-induced tumor growth by promoting *USP37* downregulation. Li et al. used bioinformatic resources (DAVID) and available datasets (GEO) to reveal that USP37 regulates CDT1 expression by deubiquitination, thereby contributing to LC tumorigenesis [31]. Furthermore, miR-320b expression and CDT1 stabilization by USP37 is inversely related. Overexpression of miR-320b downregulates *USP37* in an in vivo xenograft model, resulting in repression of the CDT1 inhibition of LC progression. New microRNAs that interact with USP37 will most likely be identified in the future and provide more information on how USP37 expression is fine-tuned.

## USP37 is a regulator of chromosomal cohesion and mitotic progression

To preserve genomic stability by proper chromosome segregation and avoid aneuploidy in daughter cells, the mitotic spindle assembly must be tightly coordinated [75]. Yeh et al. addressed the role of USP37 in chromosome cohesion and mitotic progression [76]. KD of various mitosis-associated proteins, such as phospho-histone H3, tubulin, and pericentrin, increases the mitotic index (MI) and contributes to centrosome integrity, chromosome alignment, and spindle polarity [77]. An RNAi screen in HeLa cells of 296 genes that increase the MI revealed that depletion of *USP37* markedly increases the MI, suggesting that it plays a role in mitotic progression [76]. Upon depletion of *USP37*, mitotic cells exhibit abnormal spindle morphology, chromosome misalignment, centrosome fragmentation, and multipolar spindles. Time-lapse imaging of *USP37*-depleted cells revealed centrosome fragmentation and formation of multipolar spindles, which may result from an imbalance in spindle forces. Reduced kinetochore localization of HEC1, a microtubule-binding protein at kinetochores, increases the frequency of cells in anaphase with lagging chromosomes in *USP37*-depleted cells.

Identifying USP37-interacting partners with proximity-dependent biotin identification (BioID) found that USP37 interacts with the cohesin complex proteins SMC3, SMC1, SSCC1, and SA1/2, as well with the regulators of cohesion WAPL and NIPBL [76]. WAPL is a known negative regulator of cohesin that regulates its release in prophase [78, 79]. USP37 was found to deubiquitinates WAPL. Additionally, identifying faulty kinetochore–microtubule attachments by error correction assay indicated that both USP37 and WAPL contribute to chromosomal segregation and bioriented kinetochore-microtubule attachment. *USP37*-depleted cells exhibit a six-fold increase in the number of cells in metaphase with unresolved sister chromatids, indicating that USP37 regulates sister chromatid resolution and/or chromosome cohesion. Mutations in the ubiquitin-interacting motifs (UIM) UIM2 and UIM3 of USP37 permit it to evade binding to K48- and K63-linked ubiquitin chains [80]. *USP37*-binding mutants of WAPL revealed that the UIM2 and UIM3 domains are essential for interaction with WAPL. These findings highlight a new aspect of USP37 biology, in which it specifically contributes to the stabilization of chromatin-associated WAPL through deubiquitination during mitosis, thereby regulating chromosomal segregation, cohesion and further mitotic progression.

## The curious case of USP37 function in medulloblastoma

Medulloblastoma is a poorly differentiated and hyperproliferative malignant pediatric brain tumor that predominantly arises in the cerebellum. RE1 silencing transcription factor (REST) is expressed in neural progenitor cells [81] and is an important regulator of neuronal differentiation [82, 83]. Although REST is downregulated in most differentiated neurons [81], it is aberrantly upregulated in undifferentiated pediatric medulloblastoma. Orthotopic implantation of v-MYC immortalized murine cerebellar progenitor cells that constitutively express human *REST* causes tumor formation in the mouse cerebellum [84, 85]. Notably, Das et al. reported that REST induces medulloblastoma oncogenesis by repressing *USP37* transcription, thereby leading to low levels of the p27 tumor suppressor, which controls proliferation and cell cycle exit by inhibiting CDK1 in cerebellar progenitor cells. Accordingly, USP37 is the corresponding DUB enzyme for p27 [86]. Immunoblotting of the p27 protein in REST-expressing cells reveals a laddering pattern, suggesting that p27 is highly post-translationally modified. This laddering pattern is reduced by blocking new protein synthesis with cycloheximide and rescued by proteasomal inhibitor MG132, suggesting that *REST* loss increases p27 levels by affecting its ubiquitination.

Das et al. identified four potential DUBs by investigating REST-binding *RE1* elements in gene regulatory regions and found a distal *RE1* site downstream of the gene encoding USP37. Like p27, a reciprocal association between USP37 and REST is present, and *REST* KD increases *USP37* mRNA levels. Constitutive expression of *USP37* promotes p27 deubiquitination in medulloblastoma cells, whereas a catalytically dead USP37 mutant is unable to stabilize p27.

**Fig. 4:**
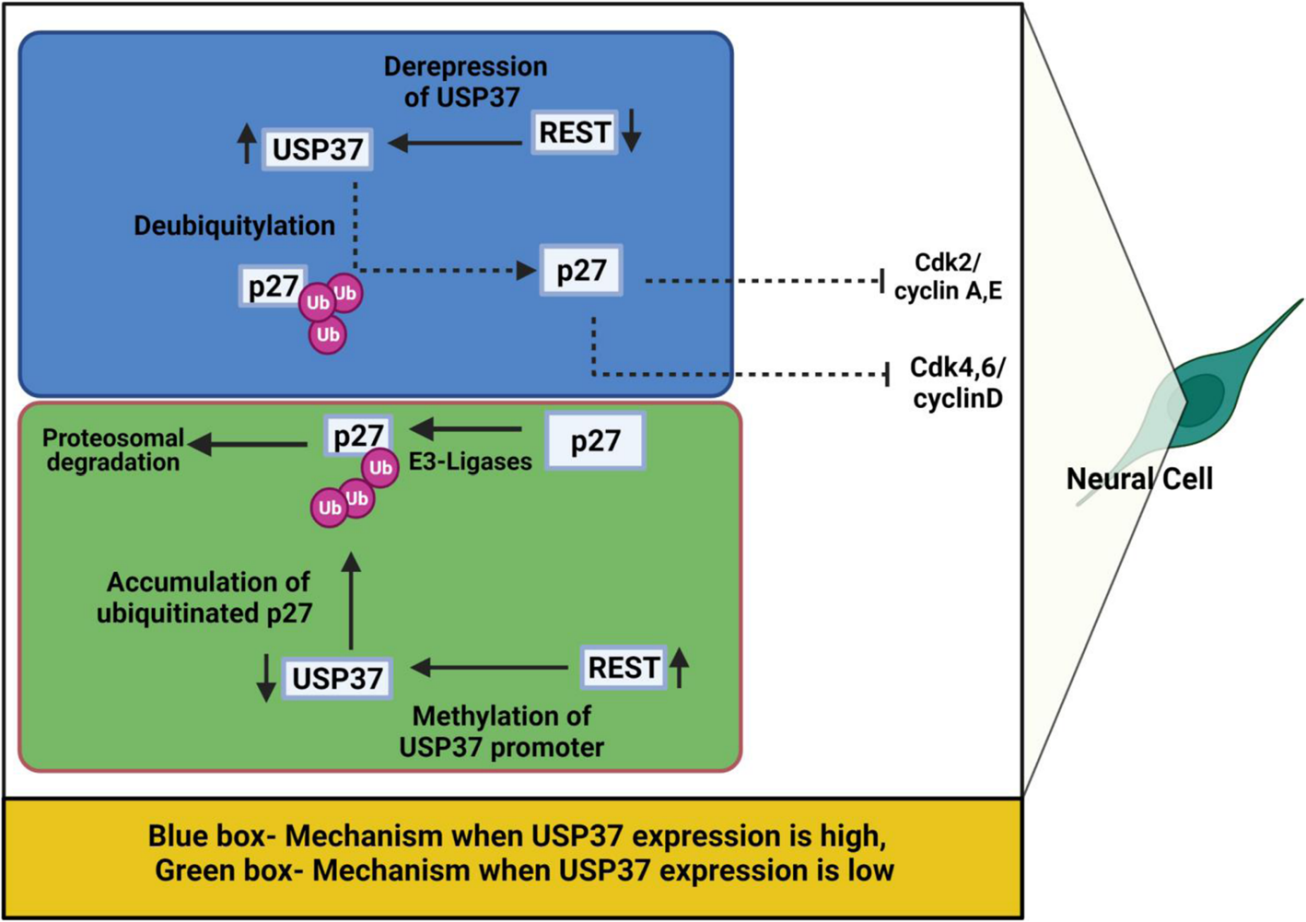
Molecular mechanism of USP37-mediated tumor suppression in medulloblastoma cells. Blue box indicates the condition during which USP37 expression is elevated due to low level of REST protein in neural cells. The Green box indicates the tumor microenvironment conditions in medulloblastoma in which USP37 activity is repressed by methylation of the *USP37* promoter due to increased REST protein levels

Dobson et al. further explored the molecular basis of REST-induced USP37 downregulation and found that USP37 supresses medulloblastoma tumor growth in an orthotopic mouse model by modulating its downstream targets [87]. Pharmacologic and genetic data from this study further provided evidence that the REST complex component G9a is involved in epigenetic regulation of *USP37*. REST globally increases G9a-dependent histone H3K9 mono-, di- and trimethylation of the *USP37* promoter, leading to downregulated *USP37* mRNA. Furthermore, inhibiting G9a activity with pharmacological inhibitors reactivate *USP37* expression, stabilizing p27 and its target genes (Fig. 4). These findings indicate that USP37 acts as a tumor suppressor in medulloblastoma, in contrast with its oncogenic function in gastric, lung, kidney, breast, and hepatic cancers.

Medulloblastoma contains a very different tumor microenvironment than that in other cancer types. Medulloblastoma is subdivided into four distinct subgroups with different clinical presentations: WNT, SHH, Group 3, and Group 4 [88]. Medulloblastoma emerges from neural cells, which are rich in neurotransmitters, such as glutamate and gamma-ammino-butyric acid, and other growth factors that may serve as optimal substrates for tumor-initiating cells (TICs) and increase survivability in the neural microenvironment [89]. USP37 function may be modulated in the neural microenvironment by some unknown modifications that promote its tumor suppressor function. Importantly, REST exhibits oncogenic activity in neural environments but has tumor suppressor activity in non-neuronal cells [90, 91]. How REST influences USP37 in non-neuronal cells is an interesting area of research. Overall, USP37 functions as a tumor suppressor in medulloblastoma, but its effects on other oncogenes and transcription factors involved in oncogenesis and cell metastasis remain unclear.

## The structural domains of USP37 and their associated interactions

**Fig. 5:**
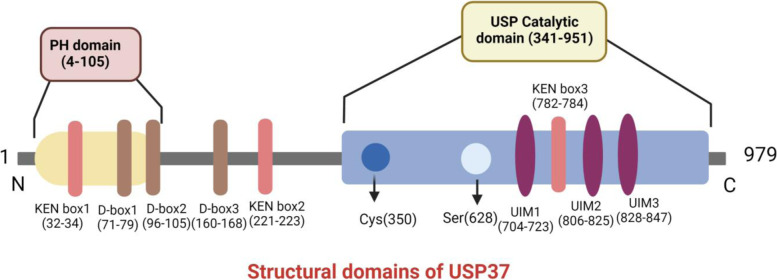
Diagrammatic representation of the structure of USP37 with its domains and critical residues required for its interaction with different oncoproteins

The studies described above shed light on the different functions of USP37 and its associated proteins and provide valuable insight into the critical domains associated with USP37 function. On the basis of findings from these studies and data available at UniProt (UniProt.org, accessed June 8, 2021), we propose a diagrammatic representation of the different domains of USP37 with critical residues (Fig. 5).

USPs commonly possess auxiliary domains flanking their core catalytic domains between conserved cysteine and histidine box motifs and often include insertions or terminal extensions that assist in the substrate and/or target recognition [92]. All USPs contain highly conserved structures resembling a right hand with three subdomains, including a finger, palm, and thumb. A cleft formed between the palm and thumb subdomains forms the catalytic core containing the catalytic cysteine and histidine residues. In contrast, the finger subdomain interacts with ubiquitin to facilitate its positioning in the catalytic center [93]. The hypothetical structure of USP37 comprises 979 amino acids residues, which consist of an N-terminal PH domain, central USP domain, C-terminal catalytic domain [94], and active sites at cysteine 350 (nucleophile) and histidine 906 (proton acceptor).

The role of cysteine 350 in USP37 was first illustrated by Huang et al. in 2011, in which they found that CDK2 activates USP37 to antagonize APC (CDH1) and promote S phase entry and that USP37 deubiquitinates CDT1 [19]. Substitution of cysteine 350 to serine abolishes its DUB activity [48]. Serine 628, which is required for full USP37 DUB activity, is phosphorylated by CDK2 during G1/S but not during mitosis. Substitution of serine 628 to alanine reduces phosphorylation by CDK2, leading to lower USP37 DUB activity [48]. The destruction box (D) domain and KEN box domain of USP37 and targets of APC and other proteases also regulate its functioning [95]. The D-box is a sequence motif found in many targets of APC with the consensus RxxLxxxxN amino acid sequence, in which x represents any amino acid. This binding pocket involves the activator WD40 domain, located in the C-terminal half of the APC/C activator subunit and a site on the APC10/DOC1 subunit of APC/C [65]. Similarly, the KEN box is a sequence motif targeted by APC with the consensus KENxxxN sequence. This motif binds the top surface of the WD40 domain of the APC activator subunit (CDH1 or CDC20) [96].

Regulation of USP37 activity through the KEN box was first demonstrated by Burrows et al., in which APC (CDH1) recognizes the KEN box-3 (782–784) of USP37 and polyubiquitinates it via K11-linked ubiquitin during late mitosis, leading to its degradation [21]. However, mutating the KEN box (782–784, KEN-AAA) impairs the interaction with APC/CDH1 and its subsequent ubiquitination [19]. In addition to the domains characterized by Huang et al., the catalytic domain contains a 284-amino acid insertion containing three Ubiquitin interacting motifs (UIMs) embedded at a site approximately 30 Å from the catalytic cleft, regulating its catalytic efficiency [19].

UIMs are single alpha-helical elements that bind to ubiquitin with modest affinity (0.1–2 mM). UIMs conform to the consensus sequence e-e-x-x-ϕ-x-x-A-ϕ-x-(ϕ/e)-S-z-x-e, in which “e” represents an acidic residue, “ϕ” represents a hydrophobic residue, and “z” represents a bulky hydrophobic or polar residue with high aliphatic content [97]. The role of UIMs in regulating the catalytic activity of USP37 was described by Tanno et al. [80]. This study demonstrated that the UIMs in USP37 are responsible for its full enzymatic activity but not its ubiquitin chain substrate specificity. Replacing alanine/valine and serine residues in each UIM with glycine and alanine, respectively, abolishes the ubiquitin-binding ability of the UIMs to proteins. The catalytic core of USP37 is inactivated by a point mutation that replaces the invariant cysteine 350 with alanine.

Detecting endogenous ubiquitin-protein conjugates that coimmunoprecipitate with transfected wild-type and UIM mutant USP37 proteins suggested only UIM2 and UIM3, rather than UIM1, permit the interaction between USP37 and ubiquitin-protein conjugates in a synergistic manner. Therefore, UIM2 and UIM3 primarily regulate the interaction of USP37 with K48- and K63-linked ubiquitin chains. The level of USP37 ubiquitination is also reduced by mutations in UIM2 or UIM3, suggesting that they play a role in the ubiquitination of USP37 itself. Examining the total cellular level of protein ubiquitination in cells expressing wild-type and mutant *USP37* revealed that UIM2 and UIM3 are required for the full catalytic activity of USP37 on endogenous ubiquitin-protein conjugates. Furthermore, UIM2 and UIM3, which are located in tandem with an eight–amino acid spacer in the region between the cysteine and histidine boxes, play an essential role in ubiquitin-binding ubiquitination of USP37, and most importantly the full catalytic activity of USP37.

Yeh et al. showed that USP37 is required for chromosome cohesion and mitotic progression [76]. Specifically, the ubiquitin-binding activities of UIM2 and UIM3 are necessary for interaction with WAPL, and mutations in UIM2 and UIM3 abolish their interaction with WAPL. UIM2 and UIM3 also contribute to the binding of ubiquitin conjugates in vitro and interaction with WAPL in vivo, further highlighting the role of UIM2 and UIM3 in the DUB activity of USP37.

A recent study by Manczyk et al. revealed that the UIMs modulate USP37 cleavage specificity and efficiency [94]. Detailed mutational, biochemical, and enzymatic characterization of USP37 UIMs elucidated the role of each UIM in chain linkage cleavage specificity for all eight possible di-ubiquitin chain types. They found that the UIMs do not confer specificity towards specific ubiquitin chain types but are instead required for USP37 full enzymatic activity. Kinetic experiments were performed with USP37 WT and mutations in all three UIMs of USP37 against the three most preferred Ub chain substrates, namely K11-, 48-, and 63-linked chains. Mutations in all three UIM domains decreased the Kcat by ~ 15-fold and increased the Km by ~ 1.8-fold for K48-linked chains, and the Kcat and Km values decreased for K11- and K63-linked chains. Therefore, the difference in the ability of the UIMs to bind ubiquitin plays a differential role on the kinetic parameters of USP37 depending on the type of ubiquitin chain, shedding light on the mechanism of substrate selection by USP37.

When individual UIM mutants were tested, the authors found that UIM2 and UIM3 modulate the ability of USP37 to cleave all ubiquitin chain types, in contrast with that of UIM1. UIM3 mainly contribute to the activity of USP37 for cleaving K11-, K48- or K63-linked chains, and in contrast, UIM1 is not requisite for the cleavage activity towards all these chains. Replacing the proximal ubiquitin moiety in a model substrate with a cleavable fluorescent molecule revealed that the UIMs specifically engage the proximal ubiquitin (primary amine donating moiety) in K48-linked chains. The UIMs also support enzymatic activity by selectively engaging the proximal ubiquitin of K48-linked chains. Furthermore, UIM2 and UIM3 selectively bind to the proximal ubiquitin of K48-linked chains to enhance cleavage by USP37, whereas UIM1 is not involved in substrate recognition [94].

Different studies revealed the role of other specific domains in different USPs and DUBs in target recognition. How USP37 is engaged in substrate selection and which domains are essential for substrate selection is an active area of research. Future domain mapping is needed to fully understand how particular domains and regions of USP37 are required for its proper functioning and stabilizing its target proteins.

## Lessons from *USP37* mRNA expression in different cancers

**Fig. 6:**
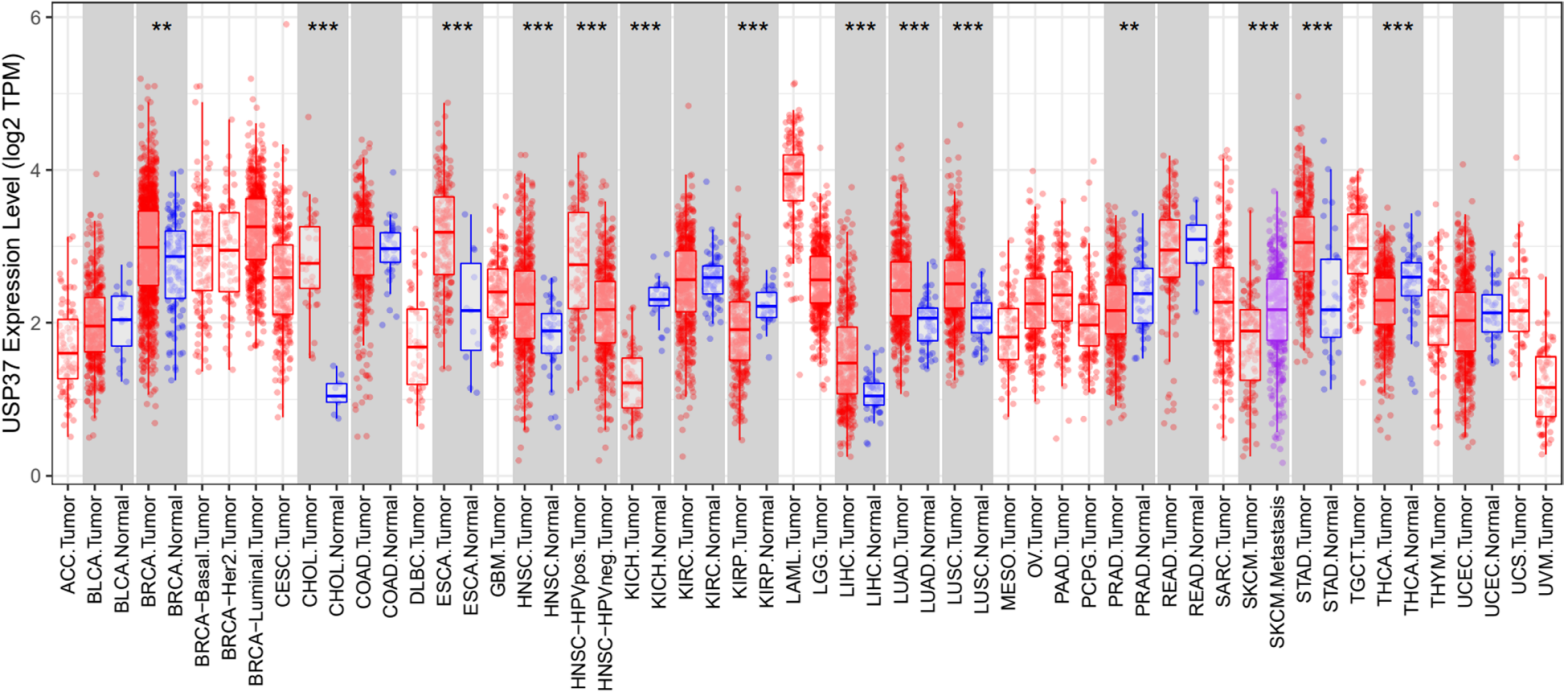
USP37 expression in different cancers from TCGA: Box plots displaying log2 Transcript Count Per Million, log2(TPM) expression of USP37 in different cancers. Red, blue, and purple bars indicate USP37 expression in normal, tumor and metastatic, tissues respectively. Significant differential USP37 expression between tumor and normal tissues or between tumor subtypes is calculated using Wilcoxon test, and the significance level is annotated by the number of stars on top of box plots (*: *p* < 0.05; **: *p* < 0.01; ***: *p* < 0.001). Tumors with no corresponding normal tissues are displayed in white columns. The cancers included are Adrenocortical carcinoma (ACC), bladder urothelial carcinoma (BLCA), breast invasive carcinoma (BRCA), cervical squamous cell carcinoma and endocervical adenocarcinoma (CESC), cholangio carcinoma (CHOL), colon adenocarcinoma (COAD), lymphoid neoplasm diffuse large B-cell lymphoma (DLBCL), esophageal carcinoma (ESCA), glioblastoma multiforme (GBM), head and neck squamous cell carcinoma (HNSC), kidney chromophobe (KICH), kidney renal clear cell carcinoma (KIRC), kidney renal papillary cell carcinoma (KIRP), acute myeloid leukemia (LAML), brain lower-grade glioma (LGG), liver hepatocellular carcinoma (LIHC), lung adenocarcinoma (LUAD), lung squamous cell carcinoma (LUSC), mesothelioma (MESO), ovarian serous cystadenocarcinoma (OV), pancreatic adenocarcinoma (PAAD), pheochromocytoma and paraganglioma (PCPG), prostate adenocarcinoma (PRAD), rectal adenocarcinoma (READ), sarcoma (SARC), skin cutaneous melanoma (SKCM), stomach adenocarcinoma (STAD), testicular germ cell tumors (TGCT), thyroid carcinoma (THCA), thymoma (THYM), uterine corpus endometrial carcinoma (UCEC), uterine carcinosarcoma (UCS), and uveal melanoma (UVM). The figure was generated with TIMER 2.0 (http://timer.cistrome.org)

We performed TCGA data analysis to examine the USP37 expression profile in different cancers (Fig. 6). We found that *USP37* is considerably upregulated in many cancers, such as invasive breast carcinoma, cholangiocarcinoma, esophageal carcinoma, head and neck squamous cell carcinoma, hepatocellular liver carcinoma, lung adenocarcinoma, lung squamous cell carcinoma, and stomach adenocarcinoma. Our TCGA data analysis correlates with observation of various studies that elevated expression of USP37 in different cancers is requisite for cell proliferation and tumorigenesis. Additional studies accentuate that various oncoproteins were stabilised by USP37 and high expression of USP37 results in poor prognosis of other cancers. Further interacting partners as in regulating USP37 and downstream targets need to be elucidated to understand the mechanistic role of elevated USP37 in various cancers.

USP37 expression is downregulated in some cancers, such as kidney chromophobe and renal papillary cell carcinoma, prostate adenocarcinoma, renal adenocarcinoma, and thyroid carcinoma, paradoxically suggesting that USP37 may behave as a tumor suppressor. Moreover, *USP37* expression is unchanged in kidney renal clear cell carcinoma. Therefore, the functioning of USP37 in different cancers is dependent upon the cancer microenvironment. Consequently, USP37 is not a bona fide oncogene or tumor suppressor though its oncogenic functions are predominantly signified by various studies. The role of USP37 in cancer progression depends on many unknown factors, and cancer-specific studies of USP37 functioning are required to understand the whole interactome and expression profiling to further mark it as an oncogene or a tumor suppressor factor in different cancers.

## Conclusion

The interplay between DUBs and E3 ubiquitin ligases in the UPS determines the activity of various proteins, which is crucial for maintaining cellular homeostasis. Deregulating the biological function of DUBs can lead to various diseases, including cancers. Efforts have been made to treat cancer by targeting UPS components in the past. Among all the UPS components targeted, the proteasome has been successfully exploited with inhibitors, such as bortezomib, carfilzomib, oprozomib, and ixazomib [98]. Improved understanding of the molecular mechanisms of DUBs in cancer provides an opportunity to develop therapeutic approaches by targeting the DUBs and/or DUB-mediated oncoprotein activity. Inhibitors have been developed for some DUBs, such as USP7, USP14, USP1, and USP9X. The biological role of USP37 and evidence of its oncogenic potential in different cancers [25, 26, 28, 39, 40, 63, 64, 70] is conclusively established (Table 1). USP37 is a tumor promoter in many cancers but also acts as a tumor suppressor in medulloblastoma [86]. This suggests that many facets of USP37 biology should be investigated by determining its different substrates in the context of different cancers because the distinct tumor microenvironment may influence its activity and substrate selection.

**Table 1:** List of USP37 specific target substrates with their mechanistic role in different cellular pathways

Pathways	Target substrate	Mechanistic role	Ref.
Cell Cycle Regulation	Cyclin A	Critical for the G1/S transition	[19, 24]
	p27	Inhibition of cell cycle progression	[86]
Oncogenesis	PLZF moiety of PLZF/RARA fusion	Cell transformation in PLZF/RARA-associated APL	[25]
	c-MYC	Cell proliferation	[26]
	14–3-3γ	Cell transformation, and promotes cell migration and invasion.	[27]
	HIF2α	Angiogenesis, glycolysis, and glucose transport and erythropoiesis.	[28]
DNA replication and DNA damage response (DDR)	BRCA1-A complex	HR pathway of DNA repair	[42]
	Cdt1	Dynamics of DNA replication	[32, 48]
Epithelial-mesenchymal transition and Metastasis	Gli 1	EMT via Hedgehog (Hh) pathway	[39, 40]
	SNAI1	Cancer cell migration and EMT mediated metastasis	[62–64]
Chromosomal cohesion and mitotic progression	Cohesin complex (SMC3, SMC1, SSCC1, SA1&2)	Chromosome’s segregation and cohesion, spindle assembly, and further mitotic progression	[76, 80]
	WAPL		

USP37 is unique because it recognizes and deubiquitinates substrates like cyclin A [19], 14–3-3 γ [27], HIF2α [28], GLI1 [41], CDT1 [48], and PLZF/RARα [25], which are not recognized by other DUBs. However, the mechanism of substrate selection and its mode of binding to different substrates are not very well understood and an active area of research. The study by Manczyk et al. demonstrated that the binding ability of USP37 UIM domains with ubiquitin depends upon the ubiquitin chain type, driving its differential interaction with ubiquitinated substrates [94]. Most importantly, how USP37 coordinates with other DUBs to fine-tune the cellular machinery by controlling the stability of proteins’ stability in oncogenic and non-oncogenic microenvironments is still unknown. Moreover, the function of USP37 in non-transformed cells is unknown so far, as all the studies reviewed here were performed with only transformed cells. A clear view of the role of USP37 in non-transformed cells is important for its development as an oncogenic target.

The role of USP37 in DDRs and regulation of replication and replication stress should be explored further to better understand its involvement in oncogenesis. Both USP37 and USP36 are involved in the HR pathway [42], but no studies defining the specific role of USP37 in DDR pathways, including NHEJ and other related pathways, are reported. Because DDR pathways are critical for drug resistance in cancers, a clear understanding of the role of USP37 in regulating these pathways may address drug resistance in some cancers. USP37 regulates DNA replication by deubiquitinating CDT1, an important protein in DNA replication, but USP37 may control many unknown factors at replication forks that should be further explored [48]. USP37 regulates replication stress by stabilizing CDT1 and CHK1, but these findings must be validated for different cancers [99].

The mechanism of activation of USP37 and the involvement of different cellular circuits in its activation is another aspect of USP37 biology that requires further scrutiny. As shown by Huang et al., phosphorylation of USP37 is required for its activation. At the transcriptional level, two tumor suppressor miRNAs in HCC and lung cancer with OSAH are involved in regulating USP37 transcription [32, 70]. A more complex mechanism and feedback loops that control cell turnover and stability are highly likely and should be a subject of future research [19]. Additional regulators of USP37 transcription will most likely be discovered in the near future, which will facilitate our understanding of the mechanisms by which *USP37* expression is regulated.

## Data Availability

Not applicable.

## Abbreviations

USP37: Ubiquitin specific peptidase 37
*SNAI1*: Snail Family Transcriptional Repressor 1
DUBs: Deubiquitinating enzymes
HR: Homologous recombination
(APC/C): The anaphase-promoting complex (APC/C)
CDKs: Cyclin-dependent kinases
APL: Acute promyelocytic leukemia
RARA: Retinoic acid receptor alpha gene
PLZF: Promyelocytic leukaemia zinc finger
PML: Promyelocytic leukaemia
MAPK: Mitogen-activated protein kinase
HIF2α: Hypoxia-inducible factor-2α
TCGA: *The Cancer Genome Atlas*
ALDH1: Aldehyde dehydrogenase isoform 1
DDR: DNA damage response
DSB: Double-strand breaks
EMT: Epithelial–mesenchymal transition
SHH: Sonic hedgehog
PLAGL2: Pleomorphic adenoma gene like-2
UIM: Ubiquitin-interacting motifs
REST: RE1 silencing transcription factor
ACC: Adrenocortical carcinoma
BLCA: Bladder urothelial carcinoma
BRCA: Breast invasive carcinoma
CESC: Cervical squamous cell carcinoma and endocervical adenocarcinoma
CHOL: Cholangiocarcinoma
COAD: Colon adenocarcinoma
DLBCL: Lymphoid neoplasm diffuse large B-cell lymphoma
ESCA: Esophageal carcinoma
GBM: Glioblastoma multiforme
HNSC: Head and neck squamous cell carcinoma
KICH: Kidney chromophobe
KIRC: Kidney renal clear cell carcinoma
KIRP: Kidney renal papillary cell carcinoma
LAML: Acute myeloid leukemia
LGG: Brain lower-grade glioma
LIHC: Liver hepatocellular carcinoma
LUAD: Lung adenocarcinoma
LUSC: Lung squamous cell carcinoma
MESO: Mesothelioma
OV: Ovarian serous cystadenocarcinoma
PAAD: Pancreatic adenocarcinoma
PCPG: Pheochromocytoma and paraganglioma
PRAD: Prostate adenocarcinoma
READ: Rectal adenocarcinoma
SARC: Sarcoma
SKCM: Skin cutaneous melanoma (SKCM)
STAD: Stomach adenocarcinoma
TGCT: Testicular germ cell tumors
THCA: Thyroid carcinoma
THYM: Thymoma
UCEC: Uterine corpus endometrial carcinoma
UCS: Uterine carcinosarcoma
UVM: Uveal melanoma
